# POETRY INTERVENTION IMPACTS PERSPECTIVES ON DEMENTIA AND CAPABILITIES AMONG YOUNG VOLUNTEERS

**DOI:** 10.1093/geroni/igz038.717

**Published:** 2019-11-08

**Authors:** Daniel B Kaplan, Gary Glazner

**Affiliations:** 1 Adelphi University, Garden City, New York, United States; 2 The Alzheimer's Poetry Project, New York, New York, United States

## Abstract

Poetry for Life (PFL), is a teaching and learning initiative that brings students together with older adults in meaningful community service workshops. PFL capitalizes on the skills and passions of young poets by offering opportunities to serve elders by leading poetry workshops at settings where older adults receive care. This study examines measurable impacts of training, exposure, and experience in poetry-based intergenerational workshops on students’ knowledge, attitudes, and values. Participating groups of students receive instruction in performing and creating poetry in group settings. They visit local elder care settings to facilitate PFL workshops and then write reflections on their experiences. Students agree to complete pre- and post-program surveys to document the impacts of PFL experiences on students' social/emotional health and on their knowledge, attitudes, and values related to older adults, dementia and dementia care, poetry and arts-based interventions, and careers in healthcare, aging fields, and the arts. To date, 33 young people from one middle school, one high school, and one graduate college program have volunteered to participate in the program and completed the study. Findings reveal significant impacts on students’ perceived capabilities working and communicating with people with dementia as well as leading poetry activities. Additionally, significant positive impacts were demonstrated on 12 of 20 items on the Dementia Attitudes Scale across participating students. The PFL experience did not, however, lead to significant impacts on student self-esteem or work interests. These findings suggest benefits and limitations of this service-learning experience. Implications for future programming will be discussed.

